# Quantitative change of EEG and respiration signals during mindfulness meditation

**DOI:** 10.1186/1743-0003-11-87

**Published:** 2014-05-14

**Authors:** Asieh Ahani, Helane Wahbeh, Hooman Nezamfar, Meghan Miller, Deniz Erdogmus, Barry Oken

**Affiliations:** 1 Cognitive Systems Laboratory, Northeastern University, Boston, MA, USA; 2 Department of Neurology, Oregon Health and Science University, Portland, OR, USA; 3 Departments of Behavioral Neuroscience and Biomedical Engineering, Oregon Health and Science University, Portland, OR, USA

## Abstract

**Background:**

This study investigates measures of mindfulness meditation (MM) as a mental practice, in which a resting but alert state of mind is maintained. A population of older people with high stress level participated in this study, while electroencephalographic (EEG) and respiration signals were recorded during a MM intervention. The physiological signals during meditation and control conditions were analyzed with signal processing.

**Methods:**

EEG and respiration data were collected and analyzed on 34 novice meditators after a 6-week meditation intervention. Collected data were analyzed with spectral analysis, phase analysis and classification to evaluate an objective marker for meditation.

**Results:**

Different frequency bands showed differences in meditation and control conditions. Furthermore, we established a classifier using EEG and respiration signals with a higher accuracy (85%) at discriminating between meditation and control conditions than a classifier using the EEG signal only (78%).

**Conclusion:**

Support vector machine (SVM) classifier with EEG and respiration feature vector is a viable objective marker for meditation ability. This classifier should be able to quantify different levels of meditation depth and meditation experience in future studies.

## Background

Mind-body medicine, such as meditation and yoga, is the most commonly used type of complementary and alternative medicine treatment [1]. MM is popular and teaches skills applicable to everyday life situations. Meditation is a practice or brain state that has been developed from many different approaches. A key facet of one meditation practice, MM, is attending to the present moment in a non-judgmental way. Specific examples of MM interventions, such as Mindfulness-Based Stress Reduction and Mindfulness-Based Cognitive Therapy, have already been formally studied and applied in a variety of clinical conditions [5-8]. However, the evidence for efficacy is not definitive [2,3] and stems from several problems including inadequate controls, inappropriate and highly variable outcome measures, lack of measures for intervention adherence, and lack of a measure that evaluates the practitioner’s ability to engage in the mind-body medicine. This latter problem is especially important. There is currently no objective measure that can assess meditation quality of the practitioner. Thus, making conclusions about mediation intervention efficacy without knowing whether the participant is actually meditating is problematic. Having an objective measure of meditation ability would greatly improve our understanding of mind-body medicine intervention trial data. Objectively assessing meditation ability might also shed light on why meditation trials succeed or fail.

Other researchers have made attempts to objectively evaluate mind-body intervention ability. Physical mind-body interventions have been able to use objective ability measures such as video recording because of their observable nature. In meditation studies, one is not able to observe the practitioners ability in the same way. Previous studies have attempted to analyze meditation ability using self-rated measures [4] although, these measures are biased by the practitioner’s self-observation. The meditation intervention literature lacks any sort of objective adherence or meditation ability measures. It is essential for the mind-body medicine field to develop an objective measure to assess meditation ability to move the field forward. Developing an objective measure to assess meditation ability may potentially be done with physiological signals that we know are sensitive to meditation such as EEG and respiration.

EEG signals may be a viable objective measure of meditation ability because they are sensitive to meditation changes. EEG changes are well-documented during meditation state changes and from long-term meditation cross-sectional trait differences [2,9-12]. EEG signals are sensitive to meditation using spectral analysis, coherence, and synchrony techniques. Some studies have found meditation state and trait-related effects on EEG voltage and power in certain frequency bands. However, which EEG component and its direction of change (greater or lesser) have not been consistently observed [9]. Spectral analysis can demonstrate the activity of brain over different regions and different states. The spectral coefficients can be employed to develop a classifier for distinguishing between meditation and control conditions, with optimal parameters, calculated on overall brain activity. Coherence, or spectral covariance, includes both amplitude and phase in a measure of phase consistency between pairs of signals in each frequency band [13,14]. EEG coherence has also been examined as an index of functional connectivity among different cortical areas in meditation studies. Increased alpha-theta range coherence has been reported both intra- and inter-hemispherically during meditation [9,15] and also as a meditation ability trait [16,17]. Although coherence does provide information on brain integration, it is limited in that it does not separate the effects of amplitude and phase in the interrelations between two signals and is thus only an indirect measure of phase locking. It is also based on Fourier analysis, which is highly dependent on the stationarity of the measured signal; this is not the case in EEG [13,18]. Other studies have examined EEG synchrony rather than just EEG spectral analysis or coherence. Synchrony is a newer approach to measuring the relationship between EEG signals recorded at different electrode sites; this measure quantifies the degree of phase-locking between different narrow band signals [13]. Gamma synchrony has been identified as a unifying mechanism in human cognitive activity [13]. One study reported increased gamma synchrony during meditation in expert meditators [19] but others have reported that most of the gamma activity recorded from the scalp is related to muscle artifact (Clinical Neurophysiology 118 (2007) 1877-1888). Alpha synchrony is associated with local and global brain networks [14]. Alpha synchrony was observed in experienced transcendental meditators compared to controls and increased alpha synchrony was seen during meditation [20]. In this study, both spectral and synchrony analysis are incorporated to develop an objective meditation marker.

EEG signals may also be potential objectives measure of meditation ability because they inform the neural correlates associated with meditation. Attending to the present moment has neural correlates but it is uncertain how specific these neural correlates are to the meditation state. Attending to the current moment in a non-judgmental way, i.e., not generating any emotional associations to what is being attended to, also presumably has neural correlates. The neural correlates of these two MM components may be subtle and the definition of meditation will improve as we learn more about the neural correlates using objective physiological markers.

In addition to EEG, respiration is another objective physiological signal that is sensitive to meditation and could be used as an objective measure. Respiration has been shown to slow down without a direct instruction to do so [21]. However, this is not consistent and likely depends on type of meditation practice. Experienced meditators often have slower respiration rates compared to controls at rest and slower minute ventilation during meditation [22]. Other meditation techniques specifically direct the practitioner to slow their breathing down [23]. Slowed breathing has known physiological effects caused by parasympathetic activation, such as decreased oxygen consumption, decreased heart rate and blood pressure, and increased heart rate variability (HRV) [23]. Slowing breathing may be a simple physiological marker within subjects to assess the degree of meditation.

The overall goal of this project was to establish an objective measure of meditation ability. This project used EEG and respiration signals as a starting point to evaluate them as objective measures of meditation ability based on their sensitivity to meditation. The EEG and respiration signals were recorded from novice meditator’s during meditation and a control condition after they had completed a six-week mindfulness meditation intervention (MMI). The recorded physiological signals were then analyzed using three quantitative methods: 1) spectral analysis of EEG signal during meditation and a control condition to determine the effect of meditation on frequency behavior of EEG data at different locations over the scalp and time-frequency analysis of respiration using Stockwell transform; 2) phase synchrony analysis using phase lock value (PLV) calculation; and 3) a support vector machine (SVM) classifier constructed to perform classification using EEG frequency coefficient, respiration signal and a joint classifier with both the EEG and respiration signal to assess the classifier ability to distinguish between meditation and control conditions.

## Methods

### Participants

The participants consisted of 28 women and 6 men, mean age 61 years (std deviation 7.6 and range 50 to 79 years). Participants were recruited with newsletters, email list serves, and flyers at Oregon Health and Science University (OHSU) and around the Portland, Oregon Metro Area. The participants were generally healthy older adults who reported stress. Inclusion criteria were: age 50–75 years old; baseline Perceived Stress Scale (PSS) [24] score ≥ 9; and willing to follow the study protocol. We focused on stressed older adults for this study because they are a large and growing population with less physiologic compensatory ability to stress than younger adults. Exclusion criteria were: evidence of cognitive impairment as assessed by a score of less than 25 on the Modified Telephone Interview for Cognitive Status (TICS) [25]; significant participant-reported medical/neurologic disease (e.g., major organ failure; insulin-dependent diabetes, active cancer, or alcoholism); significant untreated depression, as assessed by Center for Epidemiologic Studies Depression Scale (CESD) score greater than 16 and interview; were taking medications or have health condition that globally affects CNS function or physiologic measures, e.g., benzodiazepines and uremia; did not understand the instructions (e.g., cannot hear recorded instructions or are not fluent in English). These exclusion criteria were chosen to screen out participants with an underlying illness that may limit the benefit of the MMI, confound outcomes, or increase the likelihood of dropout. They also could not have prior experience with meditation classes or other mind-body classes (e.g., yoga or tai chi) within the last 24 months or more than 5 minutes daily practice in the last 30 days. The study was approved by the OHSU Institutional Review Board, and written informed consent was obtained from all participants.

### Procedure

The study design for the randomized controlled trial is as follows. All participants had a telephone screening, Visit 1 baseline assessments (week 1), six-week MMI (weeks 2–7 for immediate start group or weeks 9–14 for delayed start group), Visit 2 (week 8), and Visit 3 (week 15). Demographic data were collected during the telephone screening and Visit 1. EEG and respiration data were collected at Visit 1, 2, and 3. Following volunteer inquiries, consents were mailed for review. Interested volunteers were then screened via telephone to insure a high likelihood of eligibility. This telephone screening was approved with a Waiver of Authorization from the Institutional Review Board. Eligible participants were then scheduled for Visit 1 where eligibility was finalized, written signed consent obtained, and physiologic data collected. Participants were then randomized with a computer generated randomization program to receive the MMI immediately (immediate start group) or after week 8 following visit 2 (delayed start group). The study was single blinded: participants knew when they received the MMI but the assessor did not know whether the participants received MMI just prior to visit 2 or visit 3. Physiologic data were collected again at Visit 2. Physiologic data were then collected again at Visit 3. The data included in this report are the EEG and respiration data collected at the visit, immediately after the intervention pooled for both groups (i.e. Visit 2 for immediate start group and Visit 3 for delayed start group).

### Intervention

The specific MMI curriculum was adapted from Mindfulness-Based Stress Reduction (MBSR) and Mindfulness-Based Cognitive Therapy (MBCT) programs [26,27] and has been more fully described [28]. In brief, training included a one-on-one 60-minute session weekly for six weeks taught by a trained and experienced teacher. The in-lab sessions included three components: 1) didactic instruction and brief discussion concerning stress, relaxation, meditation, and mind-body interaction; 2) practice in meditation and other mindfulness exercises that the subjects perform both in session and daily at home; and 3) discussion about problem-solving techniques regarding their successes and difficulties in practicing and applying the exercises in daily life.

Meditation instruction included a body-awareness meditation. The instruction began with awareness of breathing and later expanded to include awareness of body sensations, and cognitive and emotional experiences. Informal exercises, such as mindful movement and mindful participation in daily activities (e.g., washing dishes), were offered to help subjects generalize mindfulness beyond the formal meditation exercises. Participants were provided with written materials and recorded audio guided meditation for each week’s at-home practice. Subjects were instructed to practice at home up to 30 minutes a day as a goal, but at least do some daily practice. The meditation homework recording had several possible shorter interval breaking points denoted by tones to allow for unpredictable time demands facing stressed adults.

### EEG recording and protocol

Physiological data were collected during two conditions 1) listening to a 15 minute National Public Radio podcast (participants chose from a list of four) with eyes closed; and 2) 15 minutes of a sitting mindfulness meditation they learned in the MMI. This meditation was guided by an audio recording as they had practiced at home. The guided meditation was used to partially match the external physical aspects of the podcast. Both recordings were done with the eyes closed. The physiologic data recorded were 32-channel EEG, EOG, respiration, ECG, and movement data was collected with movement monitors (BioSemi, Amsterdam, The Netherlands). In this study, only EEG and respiration signals were subjected to statistical analysis. The other data requires more pre-processing and different analyses but will be incorporated in the future. We used Fp1, AF3, F7, F3, FC1, FC5, T7, C3, CP1, CP5, P7, P3, Pz, PO3, O1, Oz, O2, PO4, P4, P8, CP6, CP2, C4, T8, FC6, FC2, F4, F8, AF4, Fp2, Fz and Cz channels. Raw EEG was acquired with BioSemi active electrodes using common mode sense and driven right leg electrodes just to the left and right of Cz. Linked ear channel acquired with similar referencing was also available for the analysis. Using BioSemi active electrodes, the quality of the electrode contact is assessed using offsets and the reported offsets were always in the acceptable range as stated by BioSemi. Respiration was measured with a light elastic piezoelectric belt (Ambu-Sleepmate, Maryland) around the participant’s chest near the diaphragm. Non-EEG channels were included in the meditation measure because they may improve the separation of meditation from control states.

### EEG spectral analysis

**Figure 1:**
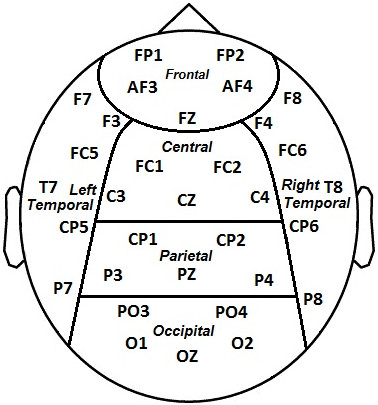
**Six regions over scalp.** Electrodes divided into six regions over scalp as frontal, central, parietal, occipital, right temporal and left temporal.

A finite impulse response (FIR) linear phase 2–35 Hz bandpass filter (Equiripple, length 2811) is applied on EEG data to avoid baseline wandering and direct current (DC) bias and high frequency noise. Two artifact removal methods were performed on the data. First a simple upper and lower voltage thresholding was used to avoid high voltages durations and flat channel effects (200 *μ*V as upper bound and 2 *μ*V as lower bound). Second, we performed an independent component analysis (ICA) to remove eye blink and eye movement artifacts using the logistic infomax ICA algorithm [29]. Subsequently, the cleaned EEG was further down-sampled to 64 Hz. It has been shown that EMG activity contributes to gamma bands recorded from scalp electrodes, therefore we have excluded gamma activity in our processing [30]. The first few samples were discarded from the data to avoid filter transient impact on signal. Power spectral density (PSD) was estimated at different electrode sites (averaged electrodes at frontal, central, parietal, occipital, right temporal and left temporal regions; Figure 1) and for different frequency bands (theta [4-8) Hz, alpha [8-12) Hz and beta [12-30) Hz). This spectral analysis was performed on data recorded during the meditation and podcast sessions.

PSD estimation is a non-parametric method utilized to calculate power spectrum of EEG signal as a function of frequency, while maintaining the balance between smoothing in time and frequency resolution. PSD is estimated using the Bartlett method [31], which averages periodograms for smoother PSD resulting in improved time/frequency resolution. This is done by dividing the data sequence into non-overlapping sequences by a Blackman-Harris window [32] and adding the resulting PSD calculated from Fourier coefficients. Let *x*[ *n*] be the signal, in our case, EEG data, divided into consecutive sequences *x*_*k*_[ *n*] for *k*=1,…,*K*, each with length *N* and *w*[ *n*] be the window function (Blackman-Harris window in this case). The resulting PSD can be estimated as:

(1) $$\hat{P}(w)=\frac{1}{K}\overset{K}{\underset{k=1}{\sum }}\left(\frac{1}{N}|\overset{N-1}{\underset{n=0}{\sum }}x_{k}[n]w[n]e^{-\text{jwn}}{|}^{2}\right)$$

Once PSD is estimated for all electrodes and frequencies, it is grouped based on location and frequency bands. The PSD values for all frequencies at each band and different locations were subjected to a two-way within subject ANOVA (analysis of variance), involving factors of Condition (Meditation, Control) and Location (Frontal, Central, Parietal, Occipital, Right Temporal and Left Temporal) (Figure 1). Before applying ANOVA the distributions were checked for normality and if any were non-normal, the Box-Cox transformation was used [33].

### Respiration time-frequency analysis

A FIR linear phase [0.01,20] Hz bandpass filter was applied to respiration data to avoid baseline wandering and DC bias and high frequency noise. A lower cut-off frequency for the low pass filter was used because of the much lower frequency content of respiration signal compared to EEG. We used time-frequency analysis (TFA) to analyze the respiration signal. TFA is a signal processing tool to study the signal and its transform jointly rather than separately. In practice, many physiological signals change their frequency content with time, while most traditional frequency transforms assume that the signal is stationary. The Stockwell transform (S-transform) [34] was used, because it reveals a wavelet-transform-like time-frequency representation of the signal and is found to be a good approach for TFA, since it employs a frequency dependent window length. Assuming *x*[ *n*]={*x*[ 0],*x*[ 1],…,*x*[ *N*−1]} be the signal, in this case the respiration signal, the S-transform is computed as:

(2) $$\begin{array}{l} \begin{array}{ll} S(\;f,n] & =A(\;f,n]e^{\mathrm{j\phi}(\;f,n]}\\ & =\overset{\infty }{\underset{m=-\infty }{\sum }}x[m]\frac{|f|}{\sqrt{2\pi }}e^{-\frac{{\left(n-m\right)}^{2}f^{2}}{2}e^{-i2\mathrm{\pi fm}}} \end{array} \end{array}$$

*S*(*f*,*n*] is the S-transform for all samples in time [ *n*] and in the frequency (*f*). Respiration data was filtered and subjected to S-transform. The time-frequency S-transform was first subjected to Box-Cox transformation [33] to form an approximately normal distribution and then was analyzed with ANOVA with a factor of condition (Meditation, Control).

### EEG phase analysis

First, baseline wandering and DC bias were removed using a FIR linear phase high pass filter with a cut-off frequency of 0.1Hz (Equiripple, length 2811). A low pass filter with a cut-off frequency of 32 Hz (Equiripple, length 260) was also applied to remove EMG noise. Then the EEG data was further down-sampled to 64 Hz. The first few samples were discarded from the data to avoid filter transient impact on signal.

We calculated phase-lock value (PLV) as a measure of synchrony for different frequency bands and between each pair of electrodes for each condition. Synchrony was calculated as such. A S-transform was used to estimate the signal phase between 4–32 Hz (The range of theta, alpha and beta bands) at 1 Hz intervals for each electrode as $\phi_{c_{i}}(\;f,n]$, *c*_*i*_ being the channel label with *i*=1,…,32. For the electrode pair (*c*_*i*_,*c*_*j*_), the phase difference can be calculated as:

(3) $$\theta_{c_{i},c_{j}}(\;f,n]=\phi_{c_{i}}(\;f,n]-\phi_{c_{j}}(\;f,n]$$

To measure synchrony, we employ *v*[ *n*], a sliding rectangular window on $\theta_{c_{i},c_{j}}(\;f,n]$ with length *L*. The PLV, a measure of how much phase synchrony exists between the two signals at the given frequency and in the given time window, was calculated as follows:

(4) $${\text{PLV}}_{c_{i},c_{j}}(\;f,n]=\frac{1}{L}|\overset{\infty }{\underset{m=-\infty }{\sum }}e^{j\theta_{c_{i},c_{j}}(\;f,n]v[n-m]}|$$

The threshold of synchrony was created by examining a distribution of PLV’s obtained from 500 pairs of surrogate signals to determine the value of PLV threshold based on what would be typically expected to occur between asynchronous signals. The surrogate signals were created by randomizing the phase of a given EEG signal, thereby destroying any synchronization of waves while maintaining identical power spectra. The histogram of all these values was derived and The significant level of *p*≥0.9 (90% confidence) is chosen to mark the high synchrony value among surrogate/random signals. This level is achieved at synchrony value equal to 0.79 on the histogram. Synchrony was defined as an electrode pair having a greater PLV value than this threshold of 0.79 at each frequency band.

The length of the window to calculate PLV was 2 seconds. Electrode pairs were arranged by taking the 6 electrodes from a region of interest and comparing them to all cross-region electrodes for a total of 76 pairs of electrodes (i.e. electrodes from the same region were not paired, because synchrony between them would be large simply due to their locations) for all times and between 4 Hz and 32 Hz. For each condition, the number of electrode pairs that had PLV above the threshold derived from surrogate signals (threshold = 0.79) were counted.

### Support vector machine classification

The first step in classification and pattern recognition studies is feature extraction. Kernel Canonical Correlation Analysis (KCCA) [35] was employed to avoid redundancy in feature vectors. KCCA measures dependency between features and it helps with decreasing the redundancy between features in feature selection.

EEG and respiration signals were processed with the S-transform and corresponding features were extracted. Letting *S*_*c*_(*f*,*n*] be the S-transform for frequency values *f*∈{ *f*_1_,…,*f*_*F*_} and time *n* for channel *c*∈{1,…,*C*} the feature vectors are:

(5) $$z_{n}={\left[z_{1n}^{T},\ldots ,z_{\text{cn}}^{T},\ldots ,z_{\text{Cn}}^{T}\right]}^{T}$$

where

(6) $$z_{\text{cn}}^{T}=\left[S_{c}(\;f_{1},n],\ldots ,S_{c}(\;f_{F},n]\right]$$

Then given {*z*_1_,…,*z*_*N*_} for one session of EEG data, the set of downsampled features were obtained using KCCA. Specifically, each feature vector for each channel was shifted sample by sample and dependency index was calculated between the original vector and the shifted version. If $e_{n}\in R^{d_{e}}$ is the signal over reproducing kernel Hilbert space (RKHS) $F^{e}$, and for *x*=*z*_*n*_ and *y*=*z*_*n*−*l*_, the *I*_*K* *C* *C* *A*_(*l*) index was computed using the following expression with appropriate substitutions.

(7) $$\;\begin{array}{l} I_{\text{KCCA}}(x,y)=\underset{g_{x}\in F^{x},g_{y}\in F^{y}}{\text{sup}}\\ \;\frac{\text{cov}\left[g_{x}(x),g_{y}(y)\right]}{\sqrt{\text{var}[g_{x}(x)+\kappa ∥g_{x}{∥}_{F^{x}}^{2}}\sqrt{\text{var}[g_{y}(y)+\kappa ∥g_{y}{∥}_{F^{y}}^{2}}}\;\;(\kappa \;>\;0) \end{array}$$

**Figure 2:**
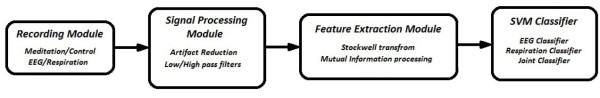
Classification process flow chart.

Then the smallest nonnegative *l*, which is a local minimizer of *I*_*K* *C* *C* *A*_(*l*) was designated as the downsampling factor *L* (i.e. *L*>0 is the smallest positive integer such that *I*_*K* *C* *C* *A*_(*L*−1)>*I*_*K* *C* *C* *A*_(*L*) and *I*_*K* *C* *C* *A*_(*L*)<=*I*_*K* *C* *C* *A*_(*L*+1)). Using *L*, the downsampled (and minimally correlated) subset of features {*z*_1_,…,*z*_1+*L*(*k*−1)_,…,*Z*_*f* *l* *o* *o* *r*(*N*/*L*)_} is obtained and has been used for the analysis below. This downsampling process ensures that we retain a large number of samples, yet minimize the statistical dependency between them to the level of the first local minimizer of the dependency measure in (8).For the EEG classifier, the S-transform was sampled along frequency in the range of [4,32] Hz with 0.25 Hz intervals. For the respiration classifier, the S-transform was sampled along frequency in the range of [0.0625,16] Hz. For the joint EEG and respiration classifier, S-transform-based feature of EEG and respiration were simply concatenated. Figure 2 shows the flowchart of the classification process. To construct a joint classifier, the feature vectors from EEG and respiration signal were combined and subjected to KCCA. All feature vectors were normalized before applying the classifier. The normalized values at each group (train, test and validation groups) were calculated by subtracting the minimum from the values and dividing the result by the range.

SVM is a supervised binary classification model that has been extremely popular in the machine learning field [36]. SVM maps the input data into a different feature space and predicts for each input data, the specific label corresponding to the output class. A discriminant function that indicates distance to the class boundary is a potential measure of meditation depth in the future.

SVM process maps the data into different categories in a higher dimensional space so that these categories are separated by an optimized margin and constructs a hyper-plane based on maximizing this margin. New data, then will be mapped into the same space and will be labeled according to their position on either side of the hyper-plane. This study used LIBSVM [37], a Matlab SVM toolbox that uses Radial basis function (RBF) kernels to nonlinearly map data into a high dimensional space.

All feature vectors were labeled as meditation or control classes and a 10-fold cross validation process was performed on the labeled data to obtain the optimized hyper parameters for the RBF kernel (width *σ*, overlap penalty *c*). Accuracy was averaged across subjects.

## Results

### EEG spectral analysis

**Figure 3:**
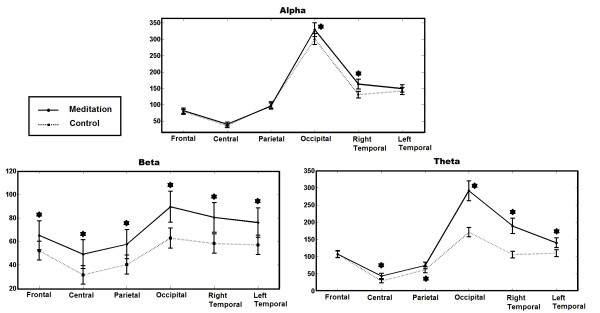
**EEG spectral analysis.** PSD estimation of EEG signal over alpha, beta and theta bands at different locations over scalp for meditation and control conditions averaged across subjects (N = 34). Star symbols show which comparisons are statistically significant.

Within each frequency band, there was a condition effect by location (alpha: *F*(5,165) = 3511, *p* ≤ 0.001, beta: *F*(5,165) = 5928.18, *p* ≤ 0.001, theta: *F*(5,165) = 2477.12, *p* ≤ 0.001). Within each frequency band, there was also a condition effect across all locations (alpha: *F*(1,33) = 10.58, *p* ≤ 0.0011, beta: *F*(1,33) = 142.03, *p* ≤ 0.004, theta: *F*(1,33) = 118.79, *p* ≤ 0.001). There is an overall increase in power during meditation in beta and theta bands, while this increase is less for alpha and is not significant in frontal region in theta. The alpha band has a slight increase in power in the right temporal and occipital locations during meditation. Figure 3 shows the effect of condition in the different frequency bands and locations and shows the value of PSD over the scalp for different conditions and different bands. Table 1 contains the numerical data corresponding to Figure 3.

**Table 1:** EEG spectral analysis results

	**Alpha**			**Beta**			**Theta**		
**Location**	**Meditation**	**Control**		**Meditation**	**Control**		**Meditation**	**Control**	
Frontal	*m*=81.93,*σ*=8.14	*m*=78.37,*σ*=7.66		*m*=65.14,*σ*=12.27	*m*=52.04,*σ*=8.04	⋆	*m*=106.99,*σ*=9.36	*m*=107.16,*σ*=10.06	
Central	*m*=40.79,*σ*=6.26	*m*=35.80,*σ*=5.80		*m*=49.10,*σ*=12.26	*m*=31.44,*σ*=7.9	⋆	*m*=43.46,*σ*=7.72	*m*=28.59,*σ*=5.16	⋆
Parietal	*m*=96.00,*σ*=9.25	*m*=99.45,*σ*=9.76		*m*=57.50,*σ*=12.38	*m*=40.12,*σ*=8.12	⋆	*m*=74.32,*σ*=9.64	*m*=61.49,*σ*=9.02	⋆
Occipital	*m*=329.13,*σ*=20.72	*m*=300.37,*σ*=16.35	⋆	*m*=89.50,*σ*=12.25	*m*=62.74,*σ*=8.63	⋆	*m*=291.44,*σ*=29.09	*m*=170.98,*σ*=13.33	⋆
R Temporal	*m*=163.18,*σ*=14.78	*m*=130.74,*σ*=10.06	⋆	*m*=80.40,*σ*=12.64	*m*=58.17,*σ*=8.27	⋆	*m*=188.96,*σ*=22.33	*m*=105.88,*σ*=9.61	⋆
L Temporal	*m*=149.46,*σ*=11.34	*m*=142.04,*σ*=10.66		*m*=76.04,*σ*=12.51	*m*=56.98,*σ*=8.25	⋆	*m*=140.19,*σ*=14.35	*m*=110.11,*σ*=10.05	⋆

EEG power for beta, alpha, and theta frequency bands in (*μV*)^2^/*Hz* (*m*= mean, *σ*= standard deviation) for control and meditation conditions. Star symbols show which comparisons are statistically significant between meditation and control states.

### Respiration time-frequency analysis

**Figure 4:**
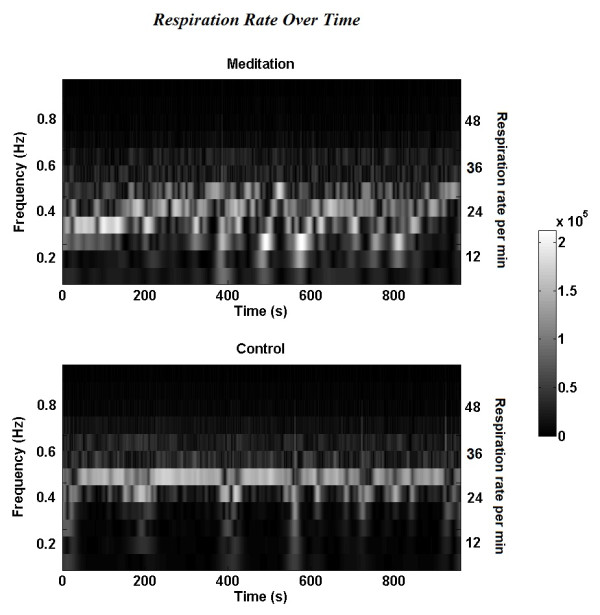
**Respiration time frequency analysis.** Time frequency Analysis of respiration signal shows more activity in lower frequencies during meditation. The figure shows the Stockwell coefficients amplitude averaged across subjects (N = 34) in meditation and control conditions.

As shown in Figure 4 there is a clear distinction between meditation and control conditions, plotted from S-transform coefficients. The lower frequencies show greater activity during meditation, while higher frequencies show greater activity during the control condition. ANOVA was applied on Stockwell coefficients involving the factor of state (Meditation, Control). ANOVAs of Stockwell coefficients resulted in a significant effect of meditation on respiration spectral coefficients (*F*(1,33) = 10.10, *p* ≤ 0.005) (Figure 4).

### EEG phase analysis

**Figure 5:**
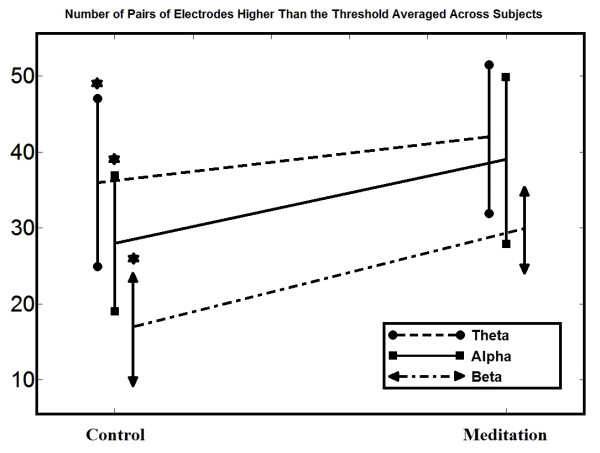
**EEG phase analysis.** Number of pairs of electrodes with higher PLV than the threshold averaged across subjects (N = 34). Star symbols show which comparisons are statistically significant.

We applied this method to data for all subjects and averaged the values as shown in Figure 5. The results indicate that the number of electrode pairs exhibiting higher synchrony is increased while subjects are meditating, relative to the control condition.

### Support vector machine classification

**Figure 6:**
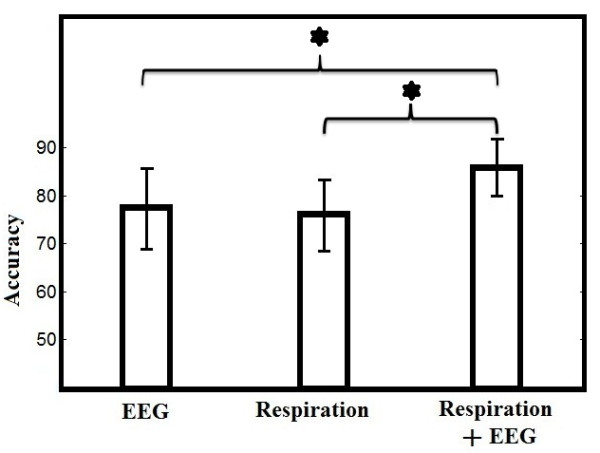
**SVM classification results.** SVM classification accuracy using EEG, Respiration and joint (N = 34). Besides the overall ANOVA being significant, all pairwise differences using paired t tests were significantly different. Star symbols show which comparisons are statistically significant.

The accuracies for EEG-only, respiration-only and joint EEG/respiration SVM classifier were averaged among subjects. Figure 6 shows the accuracy of the three classifiers. The joint classifier had a statistically higher accuracy than either the EEG or Respiratory classifiers. The accuracies of the three classifiers were subjected to repeated ANOVA and showed the effect of classifier type on accuracies (*F*(1,33) = 62.18, *p* ≤ 0.001).

## Discussion

The objective of the study was to evaluate EEG and respiration as objective measures of meditation ability. We found that among three types of SVM classifiers constructed by EEG-only, respiration-only and both feature vectors, we found the greatest accuracy using both EEG and respiration signals to discriminate between the meditation and control condition. Combined EEG and respiration signals may be a potential marker of meditation ability.

Our spectral analysis results support most, but not all, previous studies conducted on meditation on theta, alpha, and beta frequencies. The spectral analysis from 34 novice meditators revealed a generalized increase in beta and theta EEG power during meditation compared to control state (except for frontal region in theta band). This increase was smaller in the alpha band and was more focused in the right temporal and occipital locations. Increases in theta power have been widely reported during meditation. In a meta-analysis of 64 studies examining electrophysiologic measures in meditation, all of the studies that assessed theta reported an increase during meditation [9]. Other studies have demonstrated increased theta during meditation and also in expert meditators at baseline [9,15,38-41]. Additionally, the systematic review is consistent with our alpha findings. Most studies show higher alpha power during meditation relative to controls as well as cross-sectionally in expert meditators compared to non-meditators [9]. What is less common is the observation of increased beta during meditation conditions. In one study, beta and occipital alpha were increased in ten participants in a meditation versus a control condition [42]. Our study also found increased EEG synchrony in the meditation versus control condition.

Our respiration findings support previous studies that report lower respiration rates during meditation. A clear distinction between the meditation and control conditions is visible after spectral analysis of respiration data with the meditation condition having a lower respiration rate. There is heterogeneity across meditation practices in regards to regulation of breathing. Some meditation practices involve active control over inhalation and exhalation while others do not include specific instruction to alter the breath [2]. In this study, the meditation practice was a sitting mindfulness meditation practice where attention was focused on the breath, but there was no instruction to change the frequency of the breath. Other studies of mindfulness meditation have found slower breathing rates as a result of being conscious of the breath [21]. We have observed that experienced meditators had slower respiration rates compared to controls at rest and during meditation [43]. It appears that regardless of whether one is directing a slowed breathing rate during meditation, respiration is slower during meditation.

This study advances meditation research in a number of ways. First, the spectral analysis on EEG data supports most but not all existing evidence that alpha and theta EEG power are increased during the meditative state. The increase in beta band power is not as supported in the meditation literature with only a few studies documenting an increase in beta band power during various meditation types [9]. This raises the hypothesis that beta band power may be more dependent on participant experience with meditation and/or meditation type. The decrease in respiration rate during meditation is supported by other studies documenting that respiration rates often slows during meditation regardless of whether there is a specific instruction to do so or not. The lower respiration rate, may be due to the theory that meditative state overlaps with a relaxation response even though relaxation is not a directed intention of meditation [44,45]. The slowed respiration rate also provides a mechanism by which meditation reduces stress in healthy adults and multiple chronically ill populations [46,47].

This is also the first reported study to use joint SVM classifier to discriminate between meditation and non-meditation states. We found a joint EEG and respiration SVM classifier had higher accuracy in classification than EEG alone. The SVM classifier is a major step forward for the meditation research field.

There are several limitations in this study. The study used only novice meditators. A future study should include expert meditators also. For this study, in order to deal with the logistics of blinding the person recording the physiological signals, the recording during meditation preceded that during the podcast. While a sequence effect cannot be ruled out, in general, the longer the EEG recording the greater the drowsiness, which would not be consistent with the observed differences. Future studies will need to randomize or at least counterbalance the order. This study used EEG and respiration only. Future studies would include other physiological signals in the SVM classifier to ascertain the accuracy could be improved.

## Conclusion

In conclusion, our study examined EEG and respiration data during a control and meditation condition to evaluate them as potential objective measures of meditation ability. Three types of analyses were conducted: spectral analysis, phase analysis and SVM classification. The EEG spectral data support most, but not all, existing evidence that alpha and theta EEG power are increased during the meditative state and respiration rate is lower during meditation. The SVM classifier was an accurate tool for predicting meditation compared to a control state and performed better with the addition of respiration to EEG data. The SVM model with the inclusion of respiration rate and even other physiological measures might improve the classifier and could be used in other studies examining shorter time periods to determine the depth/engagement of meditation within meditation conditions. The classifier could be applied in real-time to assess meditation depth and engagement of meditation. While it may be possible to use the described SVM for real-time monitoring of meditation state, the definition of meditation state will evolve as scientists studying it use better objective markers that coarsely relate to it.

## Competing interests

The authors declare that they have no competing interests.

## Authors’ contributions

AA suggested the design of the analysis, performed the statistical analysis and was the largest contributor to the manuscript. HW assisted in the clinical study design and contributed to the manuscript preparation and editing. HN participated in statistical analysis theorem development and implementation. MM collected all the physiological data analysis and contributed to data acquisition methodology. DE participated in statistical analysis theorem development and supervised the study. BO designed the human study protocol, participated in the analysis, contributed to manuscript preparation and editing, and obtained funding for the study. All authors read and approved the final manuscript.
